# *Eucommia ulmoides* Oliv. Bark Extracts Alleviate MCAO/Reperfusion-Induced Neurological Dysfunction by Suppressing Microglial Inflammation in the Gray Matter

**DOI:** 10.3390/ijms26041572

**Published:** 2025-02-13

**Authors:** Jiarong Pan, Xuejun Chai, Cixia Li, Yongji Wu, Yue Ma, Songlin Wang, Yuhuan Xue, Yongkang Zhao, Shulin Chen, Xiaoyan Zhu, Shanting Zhao

**Affiliations:** 1 College of Veterinary Medicine, Northwest A&F University, Yangling 712100, China; 18848965572@163.com (J.P.); wyjalisa@163.com (Y.W.); mayue140@163.com (Y.M.); wangsonglin200102@163.com (S.W.); xyh921026@163.com (Y.X.); 2021050529@nwafu.edu.cn (Y.Z.); csl_1359@163.com (S.C.); 2 College of Basic Medicine, Xi’an Medical University, Xi’an 710021, China; junchai0708@126.com; 3 College of Life Science and Technology, Xinxiang Medical University, Xinxiang 453003, China; licixia@nwsuaf.edu.cn

**Keywords:** ischemic stroke, *Eucommia ulmoides* Oliv. bark, neurological recovery, neuroinflammation, network pharmacology

## Abstract

Ischemic stroke ranks as the second leading cause of global mortality. The limited time for effective thrombolytic treatment has prompted the exploration of alternative prevention approaches. *Eucommia ulmoides* (*E. ulmoides*) Oliv. bark has shown multiple pharmacological effects, including neuroprotection, anti-inflammation and autophagy modulation. This study aims to elucidate the neuroprotective effects of water extract of *E. ulmoides* (WEU) supplementation in a middle cerebral artery occlusion (MCAO) mouse model and to further explore the underlying molecular mechanisms. Seven bioactive compounds in WEU—aucubin, chlorogenic acid, geniposidic acid, quercetin, protocatechuic acid, betulin and pinoresinol diglucoside—were identified using HPLC-MS. Our results showed that WEU supplementation significantly decreased infarct volume and ameliorated neurological dysfunction in mice following MCAO/reperfusion (MCAO/R) injury. Furthermore, the administration of WEU significantly attenuated microglia activation induced by cortical ischemia in mice and inhibited the production of pro-inflammatory mediators, including interleukin-1β (IL-1β), interleukin-6 (IL-6) and tumor necrosis factor-α (TNF-α). Importantly, in contrast with the vehicle group, the protein expression levels of Toll-like receptor 4 (TLR4), phospho-p38 (p-p38) and nuclear factor kappa B (NF-κB) were reduced in the WEU group. Therefore, this present study provides evidence that *E. ulmoides* improves neurological behaviors by suppressing neuroinflammation and inhibiting the activation of the TLR4/ p38 MAPK and NF-κB pathways in mice after ischemia, which indicates that *E.ulmoides* is a promising candidate for alleviating gray matter ischemic change.

## 1. Introduction

The global incidence of ischemic stroke is increasing every year, making it the foremost cause of severe disability and mortality among adults globally [1]. The initial cause of cell death, infarct expansion, and neurological disability after ischemic stroke is the disruption of blood and oxygen supply [2]. Ischemia triggers a cascade of responses, such as excitotoxicity, oxidative stress, inflammation and mitochondrial dysfunction, that severely affect the prognosis of patients with stroke [3,4]. Recanalization by the intravenous (i.v.) administration of recombinant tissue plasminogen activator (rt-PA) alone for thrombolysis or followed by thrombectomy is the first-line treatment for patients with ischemic stroke [5]. However, the limited time window for treatment and the reperfusion-related damage present a serious clinical dilemma to physicians and scientists [6]. Therefore, it is very important to uncover new and effective therapeutic strategies for ischemic stroke to overcome treatment limitations.

Recently, network pharmacology has become a viable paradigm for the study of mechanisms of action and the discovery of potent substances from complex systems (herbal formulas, component herbs, compound preparations, etc.) [7,8]. For stroke research, network pharmacology provides a new “multi-component, multi-target, multi-pathway” network model [9]. Liu et al. [10] revealed that Xiao-Xu-Ming decoction protects against hemorrhagic transformation after ischemic stroke by exerting an anti-neuroinflammatory effect through network pharmacology combined with experimental validation. These reports have demonstrated the reliability of network pharmacology in stroke research.

*Eucommia ulmoides* (*E. ulmoides*) Oliv., a famous dicotyledonous plant belonging to the family Eucommiaceae, is used as folk medicine in Asian countries like China, Japan and Korea [11]. Traditionally, *E. ulmoides* has been credited with tonifying the kidneys and liver, strengthening bones and muscles, and repairing meridians [12]. Pharmacological experiments have proven that *E. ulmoides* possesses a sequence of therapeutic functions, such as anti-hypertensive, anti-inflammatory, immunomodulatory, anti-oxidative, anti-osteoporosis and hepatorenal protection functions [12,13,14]. Apart from the above effects, previous studies have found that *E. ulmoides* demonstrates significant neuroprotective effects in some experimental models and therefore may be a promising pharmaceutical candidate for addressing neurodegeneration [15,16,17]. Recent studies have found that *E. ulmoides* not only exhibits an anti-apoptotic effect by regulating oxidative stress but also an anti-inflammatory effect by reducing the secretion of inflammatory cytokines [18,19]. Moreover, an aqueous extract of *E. ulmoides* leaves (AEEL) alleviates DSS-induced cognitive dysfunction by regulating the gut–brain interaction [20]. However, the effect of *E. ulmoides* on alleviating the dysfunction of ischemic stroke has not been reported.

The aim of the present study is to investigate the positive roles of *E. ulmoides* in the restoration of neurofunctional and motor functions after gray matter ischemia. Network pharmacology was employed to comprehensively investigate the signaling pathways and biological processes involved in the therapeutic effects of WEU supplementation in cerebral ischemia and to validate the mechanism of WEU action in cerebral gray matter ischemic injury through molecular biology experiments. Our results showed that WEU could inhibit neuroinflammation induced by ischemic stroke by alleviating microglia activation and inhibiting the activity of the TLR4/p38 MAPK and NF-κB pathways, which indicates that *E. ulmoides* may provide new insights into potential treatment strategies for gray matter ischemic injury.

## 2. Results

### 2.1. HPLC-MS Analysis of WEU

By conducting a retention time comparison between individual peaks and standard reference materials in the HPLC-MS analysis of WEU, the identification of seven types of organic matter was achieved. They were aucubin, chlorogenic acid, geniposidic acid, quercetin, protocatechuic acid, betulin and pinoresinol diglucoside (Table 1). The mass spectra of mixed standard substances with 7 active compounds were shown in Figure S1.

### 2.2. WEU Ameliorated the Ischemia-Induced Deficits of Motor Coordination

**Figure 1:**
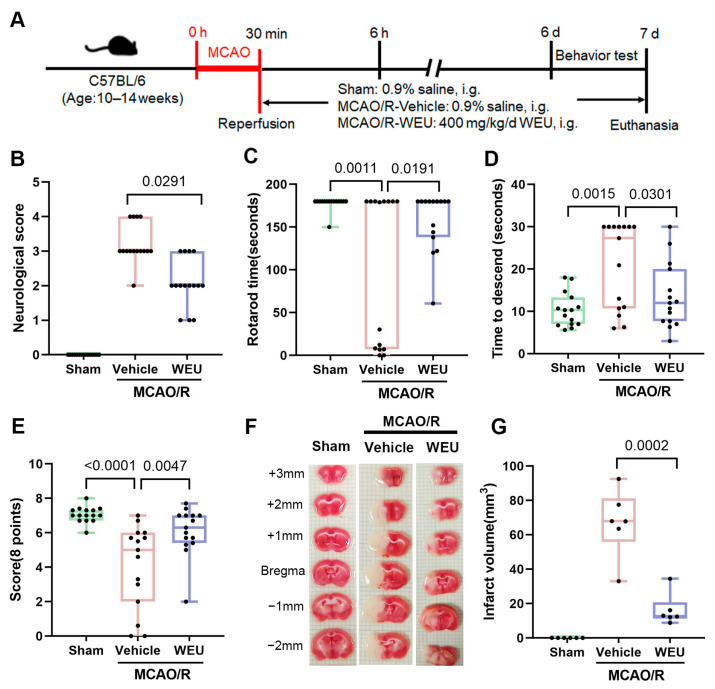
Neuroprotective effect of WEU against ischemia-induced behavioral defects and infarct volume. (**A**) Overview of the experimental design. (**B**) Neurological deficits were more remarkable in the vehicle mice than in the WEU mice (*n* = 15). Motor coordination was evaluated using the accelerated rotarod test (**C**), pole test (**D**) and grip and string tests (**E**) (*n* = 15). (**F**) Representative 1 mm thick coronal brain sections of three groups of mice stained with 2,3,5-triphenyltetrazolium chloride. Infarcted tissue appeared pale, while normal tissue was stained red. (**G**) Total infarct volumes were significantly larger in the vehicle group mice than in the WEU mice (*n* = 6). All data are presented as mean ± SEM. i.g., intragastric administration.

In accordance with the experimental design depicted in Figure 1A, the effects of WEU on post-stroke rehabilitation were evaluated by employing a combination of neurological scores and behavioral tests. Unsurprisingly, mice in the sham-operated group showed no signs of neurological dysfunction. However, the MCAO operation caused severe impairment of neurological function. In comparison with the vehicle group, the treatment with WEU contributed to a significant reduction in neurological scores of stroke mice on day 7 post-MCAO (Figure 1B).

The rotarod test and pole test were employed to detect the coordination of movement in the mice. Figure 1C illustrates that compared with the sham mice, the mice subjected to ischemia spent less time on the rotarod accompanied by impaired motor coordination. In the WEU group, the mice observably spent a longer time on the rotarod. As shown in Figure 1D, the vehicle group mice frequently displayed uncoordinated movements and dropped or slipped from the pole. In comparison with the vehicle group, the WEU group mice effectively reduced the time it took to descend from the pole and reach the ground. In addition, muscle tension was evaluated by assessing the mice’s ability to grasp in the grip and string tests. Among the three experimental groups, the mice in the vehicle group possessed impaired grasp capability, evidenced by a shorter time until falling down, while WEU supplementation obviously enhanced the capacity to grasp in the ischemic stroke mice (Figure 1E).

### 2.3. WEU Decreased Infarction Size in Post-Stroke Mice

With the aim of investigating the impact of WEU on brain infarction, a 2,3,5-triphenyl tetrazolium chloride (TTC) stain assay was conducted to calculate the brain infarction volume at 7 d following MCAO surgery. As shown in Figure 1F, the TTC-stained brain sections in the sham group exhibited a normal tissue-like uniform red color, while both the vehicle and WEU group displayed a pale color in the cerebral infarct area. Consistent with prior research results, the present study demonstrated a marked increase in the infarct volume of transient MCAO surgery mice. Fortunately, administration of WEU effectively reduced the infarct volumes induced by ischemic stroke (Figure 1G). These results suggest that WEU supplementation significantly attenuated the behavioral dysfunctions induced by ischemia.

### 2.4. Identification of WEU Anti-Brain Injury and Dysfunction Target Pathways

**Figure 2:**
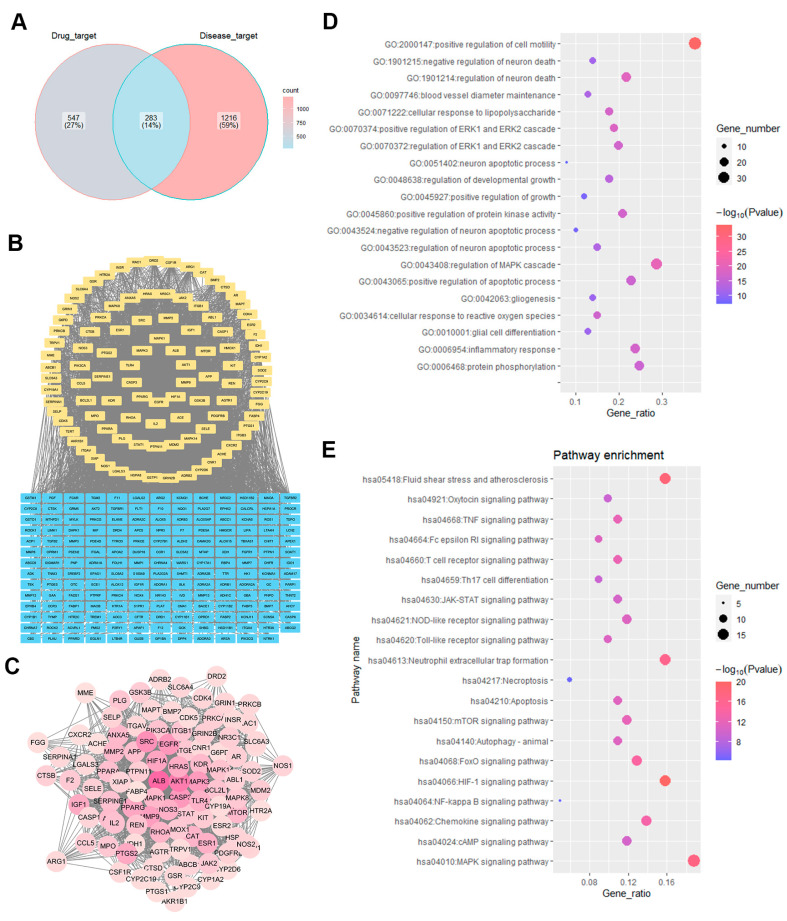
Network pharmacology prediction of WEU supplementation for ischemic stroke. (**A**) Venn diagram of overlapping target genes in the disease (ischemic stroke) and compounds (WEU). (**B**) The results of the PPI analysis for the potential targets of WEU. (**C**) The 101 core targets of WEU acting on ischemic stroke. (**D**) The top 20 biological processes were significantly enriched. The results of the GO enrichment analysis suggest that inflammatory responses may be important biological processes for WEU to promote functional recovery and mitigate brain damage. (**E**) KEGG pathway analysis of 101 target genes based on the STRING database. Top 20 significantly enriched signaling pathways were displayed according to the −log (*p*-value) scores and the number of genes.

A network pharmacology strategy was employed in this study for the exploration of the functional mechanisms responsible for the neuroprotection of WEU supplementation in relation to prognosis after stroke. By Venn analysis, 283 intersecting targets associated with both ischemic stroke and major components were screened out (Figure 2A). In total, 101 core target genes associated with the therapeutic effects of WEU on ischemic stroke were identified in 283 overlapping targets through a PPI network analysis (Figure 2B,C).

The STRING online tool was then used so that the potential mechanisms closely associated with the therapeutic impacts of WEU on ischemic stroke could be investigated. The results of a GO enrichment analysis indicated that the inflammatory response could potentially serve as the fundamental biological process involved, with relatively high number of genes and comparatively high-log (*p*-value) scores (Figure 2D). In addition, according to the findings from a KEGG pathway enrichment analysis, the MAPK signaling pathway was enriched in 19 of the 101 core target genes, while the TNF signaling pathway was enriched in 11 of the core target genes and the Toll-like receptor signaling pathway was enriched in 10 genes (Figure 2E). The above signaling pathways were closely linked to inflammation.

### 2.5. WEU Attenuated the Ischemia-Induced Generation of Pro-Inflammatory Cytokines

**Figure 3:**
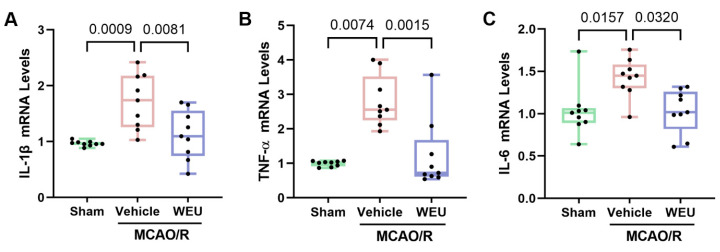
WEU attenuated inflammation in ischemic stroke mice. The mRNA levels of IL-1β (**A**), TNF-α (**B**) and IL-6 (**C**) in the cortex were assessed by Q-PCR (*n* = 9 from three mice in each group). The mRNA expressions of IL-1β, IL-6 and TNF-α were enhanced in the vehicle group. WEU supplementation effectively reduced the release of these cytokines in cortex tissues. The values are shown as the mean ± SEM. IL-1β, interleukin-1 beta; TNF-α, tumor necrosis factor-α; IL-6, interleukin-6.

To elucidate the WEU role in the regulation of inflammation after stroke, the expression levels of prominent pro-inflammatory cytokines in the cortex of mice were detected. The experiment results showed that the vehicle group exhibited a marked increase in IL-1β, IL-6 and TNF-α mRNA expression in comparison with the sham group (Figure 3A–C). Unsurprisingly, the WEU group showed a substantial downregulation of the above pro-inflammatory cytokines compared with the vehicle group. The above findings suggest that WEU suppresses the production of pro-inflammatory factors after ischemia.

### 2.6. WEU Suppressed Cerebral Ischemia-Induced Activation of Cortical Microglia

**Figure 4:**
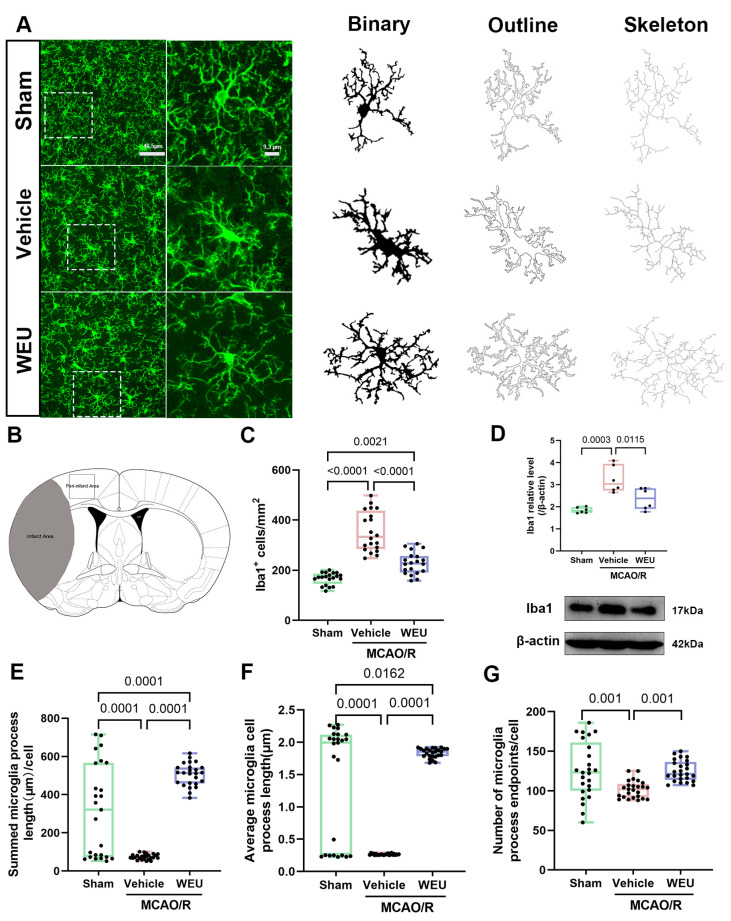
WEU alleviated microglial reactivity in the cortex of ischemic stroke mice. (**A**) Immunofluorescence images of Iba1^+^ microglia (green) in the cortex and binary, outline and skeleton images; scale = 46.5 μm or 9.3 μm. (**B**) Coronal section (Bregma 0.26) of a mouse brain showing the ischemic damage (gray area) and peri-infarct area where immunofluorescence analysis was performed, as shown in (**A**). (**C**) Quantification of the density of Iba1^+^ cells in the cortex (*n* = 9). (**D**) The verification of the quantitative analysis of the ipsilateral cortex of the mice for downregulated proteins (Iba1) by Western blot. (**E**,**F**) Statistical analysis of the number of summed microglia process lengths/cell (**E**), average microglia process length (**F**) and microglia process endpoints/cell (**G**), *n* = 25–30 per group, 25–30 Iba1^+^ cells from three mice. Data are represented as mean ± SEM.

To investigate whether WEU supplementation inhibited the focal ischemia-induced activation of peri-infarct microglia, an immunofluorescence analysis using antibodies against Iba1, which is a widely recognized marker of microglia, was employed [21]. The results showed that there was a significant elevation in the number of Iba1 immunoreactive cells after ischemia in comparison with the sham group (Figure 4C). However, WEU attenuated the Iba1-positive microglial response induced by ischemic stroke. Similar findings were observed with Western blot. Thus, WEU showed inhibitory effects on microglia activation induced by ischemia, as evidenced by the reduced Iba1 (Figure 4D). The morphology of microglia is one of their more outstanding characteristics and is closely related to their functional status [22]. To verify whether WEU inhibited the ischemia-induced microglial morphological changes, the Analyze Skeleton plugin for ImageJ was employed to measure several morphological parameters. The summed microglial process lengths per cell, average process length and endpoints/cell were substantially reduced after ischemia. Interestingly, the administration of WEU mitigated the reduction of the above microglial parameters (Figure 4E–G).

**Figure 5:**
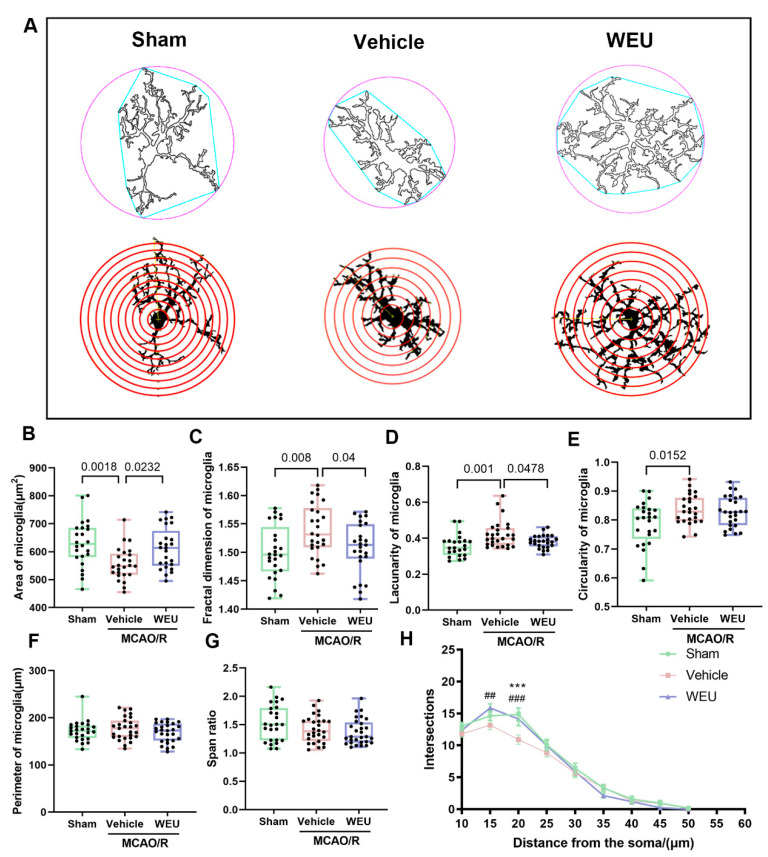
WEU reversed the morphological changes in the microglia after ischemia. (**A**) Illustration of the morphological analysis of the microglia. (**B**) Quantification of the microglial area. Cerebral ischemia increased the fractal dimension (**C**), lacunarity (**D**) and circularity (**E**) of the microglia in the peri-infarct region. However, WEU supplementation reduced the fractal dimension and lacunarity of the microglia in ischemic mice. Statistical analysis of the perimeter (**F**) and the span ratio (**G**) in the peri-infarct region. *n* = 25–30 per group, 25–30 Iba1^+^ cells from three mice. (**H**) Sholl analysis of dendrite complexity in the peri-infarct region (*n* = 25–29 per group, 25–29 Iba1^+^ cells from three mice in each group). *: sham vs. vehicle; *** *p* < 0.001. #: vehicle vs. WEU. ## *p* < 0.01, ### *p* <0.001. Data are represented as mean ± SEM.

To comprehensively assess the microglial morphological changes over the course of the inflammation, the area, fractal dimension, lacunarity, circularity, perimeter and span ratio of the microglia were measured with the free software FracLac for ImageJ (version 1.52) (Figure 5A). The analysis results showed that there was a significant decrease in the microglial area of the vehicle group compared to the sham group. WEU supplementation restored this change (Figure 5B). The fractal dimension, lacunarity and circularity of the microglia increased significantly after ischemia (Figure 5C–E). After the administration of WEU, the microglia exhibited a significant reduction in fractal dimension and lacunarity when compared to those in the vehicle group. However, there were no statistically significant variations in parameters, such as the perimeter and span ratios, among the groups (Figure 5F,G). Sholl analysis, a common approach to quantify dendritic arbor complexity in neurobiology, has been used to count the intersections between dendrites and concentric circles [23] since it was proposed in 1953 by Donald Sholl [24]. The Sholl analysis results demonstrated that the number of microglia branch crossings in the infarcted region was significantly decreased compared to the sham-operated group, especially at a shell distance of 20 μm, while the administration of WEU contributed to an elevation in the number of branch crossings (Figure 5H). Together, these findings illustrate that WEU can attenuate microglia activation and reverse the morphological changes from ischemia-induced damage.

### 2.7. WEU Inhibited the Ischemia-Induced Activation of the TLR4/p38 MAPK and NF-κB Signaling Pathways

**Figure 6:**
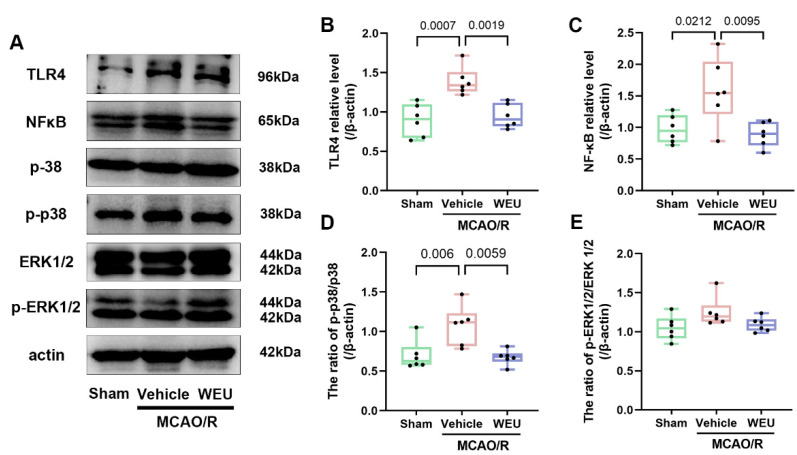
WEU inhibited the ischemia-induced activation of the TLR4/ p38 MAPK/NF-κB pathways. (**A**) Representative images of Western blotting for TLR4, NF-κB, p38, p-p38, ERK1/2 and p-ERK1/2 in the cortex of mice. Quantitative analysis of the relative levels of TLR4 (**B**), NF-κB (**C**), p-p38/p38 (**D**) and p-ERK1/2/ERK1/2 (**E**) (*n* = 6 for each group). The data are expressed as mean ± SEM. TLR4, Toll-like receptor 4; NF-κB, nuclear transcription factor kappa B; p-p38, phospho-p38; ERK1/2, p44/42 mitogen-activated protein kinase; p-ERK1/2, phosphorylated p44/42 MAP kinase.

To further elucidate whether WEU exhibits a regulatory role in inflammation pathways, inflammation-related protein expression, including TLR4, NF-κB, p38, p-p38, ERK1/2 and p-ERK1/2, were examined by Western blot in the cortical tissues of ischemic stroke mice (Figure 6A). The vehicle group exhibited a significant elevation in TLR4, p-p38 and NF-κB expression levels compared to the sham-operated group, as shown in Figure 6B–E. Interestingly, WEU administered for 7 d obviously attenuated the protein expression of TLR4 (Figure 6B), NF-κB (Figure 6C) and p-p38 (Figure 6D) compared to unadministered ischemic stroke mice. The results suggest that WEU inhibits the activation of the TLR4/p38 MAPK and NF-κB signaling pathways, attenuating neuroinflammation induced by ischemia.

## 3. Discussion

The neurological dysfunction caused by stroke seriously affects the prognosis of patients [25]. Due to its complex pathological mechanism, clinical therapeutic drugs are still lacking. Therefore, it is necessary to find new therapeutic drugs to improve the prognosis of stroke [26]. The present study showed that post-ischemic WEU supplementation had a significant gray-matter protective effect in mice with ischemic stroke. Our results demonstrate backing for the potential therapeutic effects of WEU supplementation on stroke.

The present study employed HPLC-MS to analyze the constituents of WEU. The main components were identified as aucubin, chlorogenic acid, geniposidic acid, quercetin, protocatechuic acid, betulin and pinoresinol diglucoside. Previous studies have reported the neuroprotective effects of several active components of WEU. For example, chlorogenic acid reduced 6-OHDA toxicity in SH-SY5Y cells, inhibited the production of inflammatory mediators in LPS-stimulated microglia and reduced the risk of neurodegenerative disease [27,28,29]. In addition, the administration of geniposidic acid effectively ameliorated spatial memory and cognitive function along with a reduction in cerebral amyloid-β deposition in APP/PS1 mice [30]. The above findings indicate that these active materials could potentially serve as the core compound responsible for the anti-ischemic stroke effects of WEU.

A clear clinical indicator for patients with stroke is the recovery of motor function, which can significantly improve the quality of life, and measuring this recovery in animal models is considered to be a strong indicator of therapeutic potential [31]. To systematically assess the role of WEU in the recovery of motor abnormalities due to ischemic stroke, we employed serial experimental methodology based on animal behavior research consisting of neurological deficits and pole, rotarod, and grip and string tests. Our results demonstrated that the significant enhancements in neurological function and motor coordination observed in ischemic stroke mice treated with WEU underscored its beneficial effects on recovery after post-stroke injuries. The stroke-induced high frequency of movement disorders exhibited a strong correlation with the damage to cortical motor areas and their subcortical projection pathways [32]. Interestingly, TTC staining showed that WEU supplementation contributed to a decrease in the cortical infarct volume in contrast to the vehicle group. These research findings indicate that the WEU-induced reduction of ischemia-induced cortical injury is closely associated with the recovery of motor function.

Studies indicate that as an important approach, network pharmacology has been employed widely to explore the mechanisms of action of complex components of Chinese medicine and identify potential targets of diseases [33]. Through network pharmacology research, the interrelationship among the components of TCM, therapeutic targets and diseases can be clearly explained, facilitating the elucidation of the potential targets and mechanisms of TCM [34]. To decipher the possible molecular mechanisms responsible for the pharmacological role of WEU in the restoration of ischemic stroke-induced dysfunction, the network pharmacology approach, including target prediction, PPI network analysis, GO enrichment analysis and KEGG pathway analysis, was applied. Coincidentally, the GO and KEGG enrichment analyses revealed significant enrichment in inflammatory responses and inflammation-related signaling pathways, suggesting that anti-inflammatory mechanisms are primarily responsible for the anti-stroke effects of WEU supplementation.

Accumulating evidence suggests that neuroinflammation is a major pathological process following ischemic stroke and contributes significantly to the development of brain injury, suggesting that inhibiting the development of neuroinflammation could serve as a potential treatment for ischemic stroke [35]. In ischemic stroke-induced neuroinflammation, IL-1β, IL-6 and TNF-α are three pro-inflammatory cytokines [36]. Ischemia reperfusion significantly increases IL-6 and TNF-α levels in the injured cerebral area during the subacute phase of stroke [37]. Here, we examined the mRNA expressions of IL-1β, IL-6 and TNF-α after 7 d of stroke, which are crucial for the cerebral ischemia-induced neuroinflammatory process. We found that in the brain of mice from the post-stroke vehicle group, the mRNA levels of IL-6, IL-1β and TNF-α were consistently elevated in contrast to the sham mice, while WEU reversed the abnormal contents of the above mRNA expressions in subacute stroke mice with cortical damage. The above results indicate that the positive roles of WEU in brain damage and neurobehavioral impairment after stroke were achieved in part through the modulation of the inflammatory mechanism mediated by pro-inflammatory factors. However, the improvement after WEU supplementation may be caused just by the reduced infarcted volume. Whether WEU has the same effect at the OGD cell level needs to be confirmed by further research.

Immune cells in the central nervous system (CNS) perform important macrophage functions, such as the removal of dead cells and debris and immune surveillance and response [38]. Microglia, the resident immune cells in the CNS, are responsible for the response to disturbances in functional brain homeostasis, such as ischemic stroke [39]. As is widely acknowledged, the reduced acute activation of the central myeloid cells (microglia) or the peripheral cells (monocytes and neutrophils) can attenuate stroke-induced damage [40]. As anticipated, in the mouse model, a significant increase in the number of Iba1-labeled cells (microglia) was observed. After stroke, microglia undergo significant morphological changes, from small branching structures to large amoeba-like structures, that produce pro-inflammatory factors, including IL-1β, IL-6 and TNF-α [41,42]. Quiescent myeloid microglia, moderately activated cells and highly activated microglia, especially those cells that exhibit morphological changes in response to stress, could be effectively distinguished by the imaging analysis of Iba1-positive microglia [43]. The results of the present study demonstrated that the vehicle group exhibited significant variations in microglial morphology, characterized by a reduction in the summed microglia process length, the average microglia cell process length, branching endpoints, area and Sholl branch crossings, resulting in the de-ramified microglia induced by ischemia. Notably, microglia debranching is a relevant response to microglia activation, and the amoeboid morphology caused by severe or prolonged injury indicates a complete shift to a macrophage phenotype [44]. These findings suggest that ischemia induces microglia activation. Surprisingly, the administration of WEU reversed the morphological changes induced by ischemia, indicating that WEU exerts an anti-neuroinflammatory effect by regulating microglia activation.

As a group of transmembrane pattern recognition receptors, toll-like receptors (TLRs) serve as critical regulators of cytokine production and significantly contribute to immune and inflammatory responses [45,46]. The present study demonstrated a significant elevation in TLR4 protein levels resulting from local ischemia. Multiple studies have demonstrated that the TLR4/NF-κB signaling pathway crucially facilitates the activation of neuroinflammation secondary to stroke [47,48,49], and there is evidence showing that inhibiting TLR4/NF-κB pathway activation can reduce cerebral edema and the infarct volume in rats with MCAO [50]. The MAPKs, including ERK and p38 MAPK, have a central role in stress-induced cell death as well as in apoptosis [51]. In this study, we consistently demonstrated that ischemia upregulated p-p38 and NF-κB, whereas WEU could attenuate these increases. Our results have shown that WEU promotes the inactivation of the TLR4/p38 MAPK and NF-κB signaling pathways, contributing to reduced inflammation in the presence of diseases.

## 4. Materials and Methods

### 4.1. Plant Materials and Aqueous Extract

The *E. ulmoides* bark was obtained from Tong Ren Tang Company (Beijing, China), and the WEU was obtained as described previously [52]. Boiled water (1 L) was used to extract a 100 g sample for 1 h. The first filtrate was extracted and filtered. The resulting filter residue was re-extracted twice. The combined filtrates were concentrated by heating, lyophilizing and grinding them to a powder for use.

### 4.2. HPLC-MS Analysis

The ExionLC AC HPLC system linked to a Triple Quad 4500 MS (AB SCIEX, Framingham, MA, USA) was employed to analyze the samples. The conditions of chromatography: Kinnetex C18 column (2.1 × 100 mm, 2.6 µm). For the mobile phase, 0.1% formic acid water and acetonitrile were used. The linear gradient elution is shown in Table 2, with a flow rate of 0.3 mL/min and an injection volume of 2 µL.

The conditions of mass spectrographic analysis: an ionization pattern of negative electrospray, capillary voltages, 3500 V; curation gas (CUR), 35 psi; ion source GS1, 55 psi; ion source GS2, 55 psi; electrospray voltage, −4500 V; the collision energy (CE) and declustering potential (DP) are shown in Table 3.

### 4.3. Animals and Ethical Statement

The 3-month-old male C57BL/6 mice weighing 25–30 g were provided by the Laboratory Animals Center of Xi’an Jiaotong University. The mice were kept in conditions with controlled temperature (23 ± 2 °C) and humidity (60 ± 5%), standard 12 h light/12 h dark cycle and unrestricted access to food and water. All handling of the animals was performed in accordance with the Care and Use of Laboratory Animals guidelines of Northwest A&F University.

### 4.4. Middle Cerebral Artery Occlusion (MCAO) Model

As previously described, the middle cerebral artery of the mice was occluded for 30 min [53]. In short, the mice were anesthetized with 0.56% sodium pentobarbital, the common carotid artery was separated and an arteriotomy was performed proximal to the bifurcation. The origin of the middle cerebral artery was blocked by inserting nylon monofilaments (105 μm diameter) coated with “thermomelting” glue (3 mm long, 190 μm diameter) into the internal carotid artery. After 30 min of occlusion, the filament was withdrawn for reperfusion. Body temperature was controlled at 37 ± 0.5 °C during the procedure. In the sham-operated group, the same procedure was conducted without filament insertion.

Then, the mice were placed in their cages at a temperature of 30 °C. Moistened, soft pellets were placed in petri dishes in the cage to encourage eating, and the mice were administered a subcutaneous injection of 0.5 mL of 0.9% sodium chloride immediately to avoid dehydration after reperfusion; this was repeated every 24 h for 7 d.

### 4.5. Experimental Design

Based on our previous research, we selected 400 mg/kg as the optimal concentration for further experiments [54,55]. The mice were randomly divided into three groups (n = 30 per group): a sham group, an MCAO/R injury without treatment group (vehicle) and an MCAO/R injury with WEU supplementation group (WEU). At the beginning of reperfusion, 400 mg/kg of WEU dissolved in saline was gavaged. Oral administration was repeated at 6 h after MCAO and then once daily for 7 days. The sham and vehicle groups were administrated an equivalent volume of normal saline intragastrically.

### 4.6. Assessment of Neurological Deficits

The evaluation of neurological deficits was performed on day 7 after reperfusion, as previously described [56]. The mice were scored on a scale of 0 to 5 (most severe): 0, no motor dysfunction; 1, trunk and contralateral forelimb flexion when the tail is raised; 2, rotation to the ipsilateral side with normal posture at rest; 3, rotation to the ipsilateral side; 4, roll to the ipsilateral side; 5, tilt to the ipsilateral side without spontaneous movement.

### 4.7. Behavioral Tests

#### 4.7.1. Pole Test

Previous research introduced the pole test for the assessment of motor impairment in mice [57]. A wooden pole (8 mm in diameter and 50 cm in height) wrapped with gauze to prevent the mice from slipping was vertically secured in a horizontal board. The mice were placed head down on top of the wooden pole. Then, the time taken for the mice to move from the top to the bottom of the wooden pole was calculated, and the maximum cut-off time was 30 s. Three repeated tests were conducted for each mouse, and the average value was taken.

#### 4.7.2. Rotarod Test

By conducting the accelerating rotarod test (YLS-4C, Beijing, China), balance alterations and motor coordination were detected [58]. The day before the test, the mice were placed on the rod with their heads facing forward and habituated at a constant speed of 10 rpm/min for 3 min. If a mouse fell halfway, it was put back, ensuring that each mouse had run for 3 min. On the test day, the mice were tested at 10 rpm/min, with a maximal termination time of 3 min. The mean duration of the three trials was used to represent the functional recovery of the mice.

#### 4.7.3. Grip and String Tests

A previous study recorded the process of the grip and string tests [59]. Briefly, the mice were placed between horizontal metal rods (60 cm long and 40 cm high). The length of time they remained on the rope in some way (that is, using one to four paws, the tail or paws plus tail) for 30 s was measured. The scores of the grip and string tests were summed, with a maximum of 8 points.

### 4.8. Calculation of Infarct Volume

All the mice were sacrificed 7 d after MCAO for lesion volume analysis. After death, the brain was taken and dissected into 1 mm coronal sections. Then, 2% 2, 3, 5-triphenyl tetrazolium chloride (Sigma, Tokyo, Japan) was used to stain the sections at room temperature for 30 min in the dark. Subsequently, the sections were soaked in 4% paraformaldehyde (PFA) for 1 h, arranged in order and photographed. The 2, 3, 5- triphenyl tetrazolium chloride solution is a mitochondrial function marker that can reliably indicate ischemic areas within 7 d after ischemia [60]. The total infarct volume of the damaged parenchyma was quantified by ImageJ software (version 1.52), as described by Fréchou M et al. (2020) [61].

### 4.9. Network Pharmacology Analysis

The chemical composition information of *E. ulmoides* was gathered from the Traditional Chinese Medicine Systems Pharmacology (TCMSP, https://old.tcmsp-e.com/tcmsp.php, accessed on 18 March 2023) database. Compounds with oral bioavailability (OB) ≥ 30% and drug affinity (DL) ≥ 0.18 were collected for further study based on the pharmacokinetic parameters of absorption, distribution, metabolism and excretion (ADME). Potential targets for these selected compounds were obtained from the PharmMapper (http://lilab-ecust.cn/pharmmapper/submitfile.html, accessed on 18 March 2023) website and Swiss Target Prediction (http://swisstargetprediction.ch/, accessed on 18 March 2023) website.

Targets related to “Ischemic Stroke” were provided by the OMIM database (https://www.omim.org/, accessed on 22 March 2023), DrugBank database (https://www.drugbank.ca/, accessed on 22 March 2023) and GeneCards database (https://www.genecards.org/, accessed on 22 March 2023) [62,63,64].

Protein–protein interaction (PPI) analysis was conducted by importing compound–disease interaction target genes into Cytoscape 3.8.0 software and selecting genes with a degree, betweenness centrality and closeness centrality greater than their respective medians as core target genes. The enriched Gene Ontology (GO) terms and the Kyoto Encyclopedia of Genes and Genomes (KEGG) pathway enrichment analysis were carried out using the STRING (https://string-db.org/, accessed on 25 March 2023) website, visualized using R (Version 4.0.2).

### 4.10. RNA Extraction and Real-Time Quantitative PCR

TRIzol reagent (Invitrogen, Lafayette, CO, USA) was used to extract total RNA from the mouse cortex, which was subsequently reverse transcribed to cDNA through the FastKing RT kit (TIANGEN Biotech, Beijing, China). q-PCR was performed by the SYBR^®^ Green Premix Pro Taq HS qPCR Kit (Accurate Biotechnology (Hunan) Co., Ltd., Changsha, China) and analyzed with a real-time system (Bio-Rad, Hercules, CA, USA). Mean cycle threshold (Ct) values measured in triplicate along with being normalized to an endogenous reference (GAPDH) were used to calculate gene levels. The following are the specific primers: TNF-α, F: 5′-AGTCCGGGCAGGTCTACTTT-3′, R: 5′-GTCACTGTCCCAGCATCTTGT-3′; IL-1β, F: 5′-TGACGGACCCCAAAAGATGA-3′, R: 5′-TCTCCACAGCCACAATGAGT-3′; IL-6, F: 5′-ACCGCTATGAAGTTCCTCTC-3′, R: 5′-CTCTGTGAAGTCTCCTCTCC-3′; GAPDH, F: 5′-AGGTTGTCTCCTGCGACTGCA-3′, R 5′-GTGGTCCAGGGTTTCTTACTCC-3′. The relative mRNA expressions were determined by the 2^−ΔΔCt^ method [65]

### 4.11. Immunofluorescence Staining

After being deeply anesthetized, the mice were treated with cardiac perfusion with 0.9% saline followed by 4% paraformaldehyde (PFA, pH 7.4). Coronal sections (thickness of 50 μm) were prepared from the fixed brain tissues using a vibratome (VT 1000S, Leica, Wetzlar, Germany). Sections were incubated overnight at 4°C with rabbit anti-ionized calcium binding molecule 1 (Iba1, ab178847, Abcam, Cambridge, UK) diluted in blocking solution (4% BSA, 1% NGS, 0.3% Triton in phosphate buffer). The next day, all sections were rinsed five times (8 min per wash) in PBS and incubated with a second antibody, Alexa Fluor 488 goat anti-rabbit IgG (ab150077, Abcam, UK), at room temperature for 2 h. Cell nuclei were counterstained with DAPI at room temperature for 20 min. The sections were rinsed in PBS five times again and embedded with a fluorescence mounting medium (DAKO, Carpentaria, Australia).

### 4.12. Microglia Analysis

To quantify the density of the Iba1-labeled microglia in the infarction border zone, a structured illumination microscope (Axio Observer Z1, Zeiss, Oberkochen, Germany) was used to obtain images. Then, ImageJ Fiji software (version 1.52) was used to count the Iba1-positive cells. To quantify the morphological changes of the microglia, a confocal laser microscope (TCS SP8, Leica) with 50× magnification and an image matrix of 1024 × 1024 pixel was used to collect images. Multi-planar virtual Z-mode allowed 11 images (2 μm thick) to be acquired in tissue sections at a depth of 22 µm, which were then combined to provide individual high-quality images, including detailed magnification of cell branching. To quantify the morphological changes of the microglia cells over the course of the inflammation, three plugins for ImageJ Fiji free software, for Sholl analysis, Skeleton analysis and Fractal analysis, were employed, in accordance with previous studies [66,67].

### 4.13. Western Blot

The ipsilateral cortex of the mice was separated and preserved at −80°C. Previous research described the procedure of the Western blot analysis [68]. Briefly, the samples were homogenized in ice-cold RIPA buffer (Solarbio, Beijing, China) consisting of 1% phosphatase inhibitors (Roche, Basel, Switzerland) and phenylmethanesulfonyl fluoride (Solarbio, Beijing, China). An equivalent quantity of proteins (20 μg) was isolated on 10% sodium dodecyl sulfatepolyacrylamide gel electrophoresis and subsequently transferred to a polyvinylidene difluoride (PVDF) membrane (Millipore, Darmstadt, Germany). Then, 5% skimmed milk was made to block the membranes at room temperature for 2 h. After that, the membranes were incubated with the following primary antibodies at 4°C overnight: rabbit anti-TLR4 (#ab13556, 1:1000), rabbit anti-NF-κB (#ab16502, 1:1000) (purchased from Abcam (Cambridge, MA, USA)); rabbit anti-p38 (#9212S, 1:1000), rabbit anti-phosphorylated p38 (p-p38, #4511S, 1:1000), rabbit anti-p44/42 MAP kinase (ERK1/2, #9102S, 1:1000) and rabbit anti-phosphorylated p44/42 MAP kinase (p-ERK1/2, #4370S, 1:1000) (purchased from Cell Signaling Technology (Danvers, MA, USA)). After being rinsed with TBST, the membranes were incubated at room temperature for 2 h with horseradish peroxidase-conjugated goat anti-mouse IgG antibody or goat anti-rabbit IgG antibody (1:5000, Cell Signaling Technology (Danvers, MA, USA)) and imprinted with an enhanced chemiluminescence (ECL) detection kit (GE Healthcare, Buckinghamshire, UK). Finally, ImageJ software was employed for the densitometric analysis.

### 4.14. Statistical Analysis

Statistical analysis was carried out with the assistance of GraphPad Prism v.8.0 software (San Diego, CA, USA). All data were calculated as mean ± standard error (SEM). One-way analysis of variance (ANOVA) followed by a Tukey test was conducted to evaluate statistical significance. For the Sholl analysis, repeated data were measured using two-way ANOVA followed by a Tukey test. The nonparametric Kruskal–Wallis test and post hoc Dunnett’s multiple comparison test were used to analyze the neurological scores, and *p* < 0.05 was considered to be statistically significant. These analyses were carried out by researchers unaware of the research methodology.

## 5. Conclusions

In summary, the current study suggests that the administration of WEU in the subacute phase of ischemic stroke facilitates functional recovery after stroke by alleviating cortical inflammation. WEU inhibited brain injury induced by ischemia by attenuating microglia activation and pro-inflammatory cytokine release. Furthermore, the anti-inflammatory effect of WEU was closely linked to its ability to regulate the morphological changes of the microglia and inhibit TLR4/p38 MAPK and NF-κB pathway-mediated inflammatory reactions. Collectively, the above results pave the way for a novel direction in developing a therapeutic strategy for ischemia stroke and indicate that WEU may be used as a new candidate target and idea for the development of drugs to treat ischemic stroke.

## Figures and Tables

**Table 1:** Concentrations of seven active compounds in WEU.

Compounds	Standard (µg/L)	WEU (µg/L)
aucubin	192	166
chlorogenic acid	208	17,900
geniposidic acid	185	1360
quercetin	215	160
protocatechuic acid	146	18.6
betulin	185	167
pinoresinol diglucoside	185	86.4

**Table 2:** Procedure conditions for gradient elution.

Table	A%	B%
0	97	3
3	97	3
7.5	85	15
12	85	15
18	40	60
21	10	90
22	10	90
23	97	3
25	97	3

**Table 3:** Parameters of mass spectrometry.

Compound	DP (V)	CE (V)
Aucubin	−30	−13
Chlorogenic acid	−70	−20
Geniposidic acid	−85	−26
Quercetin	−40	−12
Protocatechuic acid	−15	−20
Betulin	−180	13
Pinoresinol diglucoside	−30	−34

## Data Availability

The data presented in this study are available on request from the corresponding author.

## Supplementary Materials

The supporting information can be downloaded at: https://www.mdpi.com/article/10.3390/ijms26041572/s1.
