# Supplementary material for: Eucommia ulmoides Oliv. Bark Extracts Alleviate MCAO/Reperfusion-Induced Neurological Dysfunction by Suppressing Microglial Inflammation in the Gray Matter
Source: Int J Mol Sci. 2025 Feb 13;26(4):1572. doi: 10.3390/ijms26041572 (PMC11855810; doi:10.3390/ijms26041572)
Supplement: Supplementary file 1 [file ijms-26-01572-s001.zip › ijms-3418076-supplementary.pdf]

Supplementary material

Figure S1. Mass spectra of mixed standard substances with 7 active compounds.

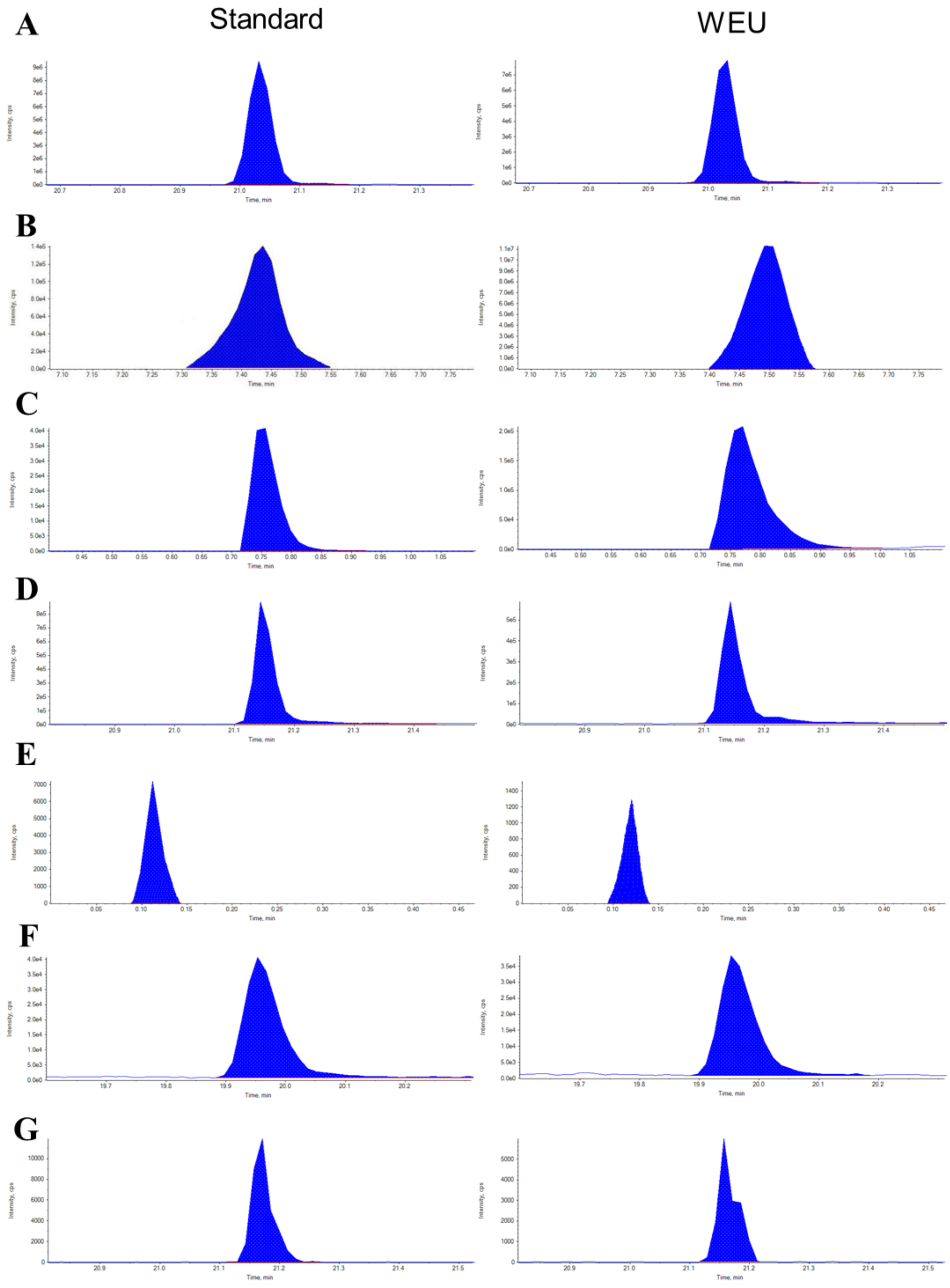

A: aucubin, B: chlorogenic acid, C: geniposidic acid, D: quercetin, E: protocatechuic acid, F: betulin, G: pinoresinol diglucoside.
